# Lost in translation: returning germline genetic results in genome-scale cancer research

**DOI:** 10.1186/s13073-017-0430-4

**Published:** 2017-04-28

**Authors:** Amber L. Johns, Skye H. McKay, Jeremy L. Humphris, Mark Pinese, Lorraine A. Chantrill, R. Scott Mead, Katherine Tucker, Lesley Andrews, Annabel Goodwin, Conrad Leonard, Hilda A. High, Katia Nones, Ann-Marie Patch, Neil D. Merrett, Nick Pavlakis, Karin S. Kassahn, Jaswinder S. Samra, David K. Miller, David K. Chang, Marina Pajic, Australian Pancreatic Cancer Genome Initiative, John V. Pearson, Sean M. Grimmond, Nicola Waddell, Nikolajs Zeps, Anthony J. Gill, Andrew V. Biankin

**Affiliations:** 1 Cancer Research Program, Garvan Institute of Medical Research, Kinghorn Cancer Centre, Sydney, Australia; 2 St John of God Subiaco, Perth, Australia; 3 School of Surgery, The University of Western Australia, Perth, Australia; 4 Cancer Diagnosis and Pathology Group, Kolling Institute of Medical Research, Royal North Shore Hospital, Sydney Australia and University of Sydney, Sydney, Australia; 5 Department of Surgery, Royal North Shore Hospital, Sydney, Australia; 6 QIMR Berghofer Medical Research Institute, Brisbane, Australia; 7 Hereditary Cancer Clinic, Prince of Wales Hospital, Randwick, Sydney, Australia; 8 Genetic and Molecular Pathology, SA Pathology, Women’s and Children’s Hospital, North Adelaide, Adelaide, Australia; 9 Wolfson Wohl Cancer Research Centre, Institute of Cancer Sciences, University of Glasgow, Glasgow, Scotland UK; 10 West of Scotland Pancreatic Unit, Glasgow Royal Infirmary, Glasgow, UK; 11 Department of Surgery, Bankstown Hospital, Eldridge Road, Bankstown, Sydney, Australia; 12 South Western Sydney Clinical School, Faculty of Medicine, University of New South Wales, Liverpool, Australia; 13 St Vincents Hospital, Darlinghurst, Australia; 14 Western Sydney University Clinical School, Sydney, Australia; 15 Division of Surgery, School of Medicine, Western Sydney University, Sydney, Australia; 16 Cancer Genetics Department, Royal Prince Alfred Hospital and Liverpool Hospital, Sydney, NSW Australia; 17 Sydney Cancer Genetics, Sydney, Australia; 18 Genetics Department, SEALS Pathology, Prince of Wales Hospital, Randwick, Sydney, Australia; 19 School of Medicine, University of New South Wales, Sydney, Australia; 20 Department of Medical Oncology, Royal North Shore Hospital and Faculty of Medicine, University of Sydney, Sydney, Australia; 21 University of Melbourne, Parkville, Australia; 22 Illumina Inc, 5200 Illumina Way, San Diego, CA 92122 USA; 23 Wolfson Wohl Cancer Research Centre, Institute of Cancer Sciences, University of Glasgow, Garscube Estate, Switchback Road, Bearsden, Glasgow, UK

**Keywords:** Genomic data, Return of results, Research ethics, Whole-genome sequencing

## Abstract

**Background:**

The return of research results (RoR) remains a complex and well-debated issue. Despite the debate, actual data related to the experience of giving individual results back, and the impact these results may have on clinical care and health outcomes, is sorely lacking. Through the work of the Australian Pancreatic Cancer Genome Initiative (APGI) we: (1) delineate the pathway back to the patient where actionable research data were identified; and (2) report the clinical utilisation of individual results returned. Using this experience, we discuss barriers and opportunities associated with a comprehensive process of RoR in large-scale genomic research that may be useful for others developing their own policies.

**Methods:**

We performed whole-genome (n = 184) and exome (n = 208) sequencing of matched tumour-normal DNA pairs from 392 patients with sporadic pancreatic cancer (PC) as part of the APGI. We identified pathogenic germline mutations in candidate genes (n = 130) with established predisposition to PC or medium–high penetrance genes with well-defined cancer associated syndromes or phenotypes. Variants from candidate genes were annotated and classified according to international guidelines. Variants were considered actionable if clinical utility was established, with regard to prevention, diagnosis, prognostication and/or therapy.

**Results:**

A total of 48,904 germline variants were identified, with 2356 unique variants undergoing annotation and in silico classification. Twenty cases were deemed actionable and were returned via previously described RoR framework, representing an actionable finding rate of 5.1%. Overall, 1.78% of our cohort experienced clinical benefit from RoR.

**Conclusion:**

Returning research results within the context of large-scale genomics research is a labour-intensive, highly variable, complex operation. Results that warrant action are not infrequent, but the prevalence of those who experience a clinical difference as a result of returning individual results is currently low.

**Electronic supplementary material:**

The online version of this article (doi:10.1186/s13073-017-0430-4) contains supplementary material, which is available to authorized users.

## Background

The advent of high throughput and more rapid genomic technologies offers an extraordinary opportunity to further our understanding of the contribution of genetic variation in complex diseases such as cancer. Considerable international efforts spanning the past decade have mapped the genomes of large cohorts of cancer patients and provided insights into prevention, early detection and treatment of cancer [1, 2]. These efforts continue to accelerate rapidly on a technical, scientific and methodological level with increased capacity and breadth of cases studied. Such work is shedding new light on what effect these genetic events have on clinical phenotypes, such as how specific genetic features relate to patterns of metastasis and treatment response. Although comprehensive catalogues of mutations across a wide range of cancers has provided us with foundational knowledge, considerable challenges remain with respect to implementing this knowledge within existing clinical frameworks.

Whether it is appropriate to give individually relevant research results back to participants in research studies, or so-called return of individual results (RoR), has emerged broadly as a complex and contentious issue that remains fervently debated [3–8]. Nevertheless, several jurisdictions have agreed that genomic results, which meet scientifically accepted criteria, should be disclosed through appropriately developed and approved frameworks within a research context [9–11]. In addition, it has been reported in the cancer setting that participants express great desire to have important research results reported to them directly and also to their relatives and spouses if results become available after the participant is deceased [12]. The myriad commentaries and reports describing approaches to RoR have focused on key upstream components such as the interpretation and classification of variants for clinical significance, the frequency and types of findings that should be returned, and with whom the responsibility of returning results lies. Despite the debate, actual data related to the experience of giving individual results back, and the impact these results may have on clinical care and health outcomes, are sorely lacking. Furthermore, it has consistently been commented that policy for RoR in research settings is required and there is a call for real approaches and outcomes of RoR to be widely shared, compared and evaluated [3, 9].

The RoR process is further complicated within large-scale genome research where the premise of such research involves the sharing of samples and data across international borders and broad accessibility of these large datasets to the research community. The diverse legal, ethical and cultural norms implicit in international studies requires careful navigation across different jurisdictions and therefore harmonisation is a vital goal.

Through the prospective observational cohort design of the Australian Pancreatic Cancer Genome Initiative (APGI), we present our real-world experience of the RoR process in a contemporary research setting, detailing the trajectory from recruitment to clinical utilisation. Using these data, we aim to: (1) delineate the pathway back to the patient where actionable research data were identified in the germline; and (2) report the clinical utilisation of individual results returned. Through this we will explore the barriers and opportunities associated with a comprehensive process of RoR in large-scale international genomic research and provide a commentary on how the pragmatic approach adopted in Australia can be potentially generalised to other jurisdictions.

## Methods

### Study population

Patients were recruited through participating institutions of the APGI between 2009 and 2013 (http://www.pancreaticcancer.net.au) as part of the International Cancer Genome Consortium (ICGC; http://www.icgc.org). Samples were contributed by third-party international collaborators as part of the Pancreatic ICGC effort. Prospective participants were provided with information that detailed the possibility of finding genetic variations that may be of relevance to them or their family members and that such findings may not be limited to their diagnosis of pancreatic cancer (PC). The consent process allowed participants the choice to ‘opt in’ to have individual results communicated, and given the short survival of many individuals with PC, the choice of to whom else they may be communicated. Ethical approval was obtained from the human research ethics committee at each participating site. All participants provided written informed consent upon entry to the study.

### Pathway for RoR process

**Fig. 1:**
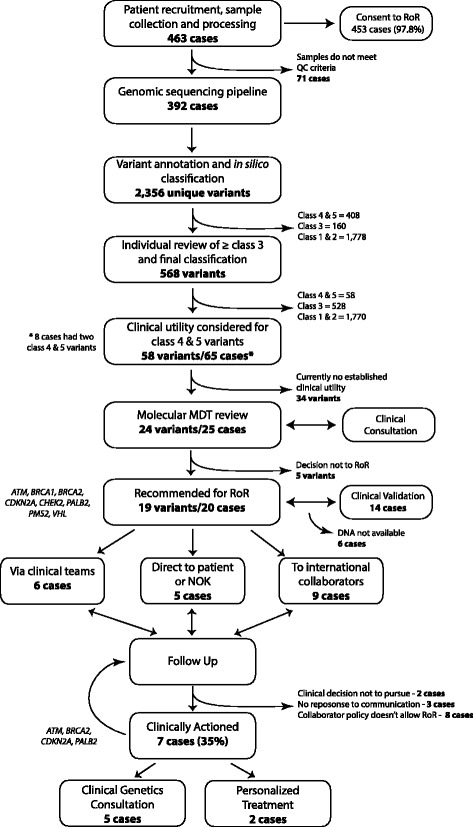
Return of results workflow

The Australian National Statement on ethical conduct in Human Research (2007) section 3.5.1 states ‘Where research may discover or generate information of potential importance to the future health of participants, or their blood relatives, researchers must prepare and follow an ethically defensible plan to disclose or withhold that information’. Given this obligation in Australia, a previously described framework was utilised in this study [13], which employs a context-dependent approach and enacts a category-based system for the characterisation of research findings as endorsed by others [14, 15]. The central components of the framework are informed consent, clinical utility, multidisciplinary (MDT) review, communicability and follow-up. Communicability considers the practicality of communicating results, the circumstances of the participant and treating clinician and how best the results could be communicated. Wherever allowable, results are to be communicated to the clinical care provider or treating medical team for their consideration (Additional file 1). The overall process is outlined in Fig. 1. Candidate genes were selected after review of the published literature and the Online Mendelian Inheritance in Man (OMIM) database. The list (n = 130, Additional file 2) includes genes with established risk for PC, genes with a cancer-related syndrome or a well-characterised solid organ or haematological cancer phenotype [6, 16, 17]. Considering both the practical and ethical aspects, the focus was strictly on cancer-related genes as these were related to the research indication. This framework was approved by each institutional Human Research Ethics Committee and data recorded by research personnel at each step (Fig. 1).

### Interpretation and classification of variants

The genomic coordinates of germline variants were annotated for gene consequence with ensembl v75 [18]. The effect of missense variants was predicted using polyphen2 [19], SIFT [20], CADD [21], VEST3 [22], alignGVGD [23], MutationTaster [24], phyloP [25] and phastCons [26]. Allele frequency in the general population was obtained from the 1000 Genomes Project (http://www.internationalgenome.org/), Exome Aggregation Consortium (http://exac.broadinstitute.org/about) [27] and dbSNPv141 (http://www.ncbi.nlm.nih.gov/SNP). The results were compiled and variants ranked using a five-tiered schemata adapted from international guidelines [28, 29] (class 5 = pathogenic, class 4 = likely pathogenic, class 3 = uncertain significance, class 2 = probably no pathogenicity, 1 = no pathogenicity, Additional file 3). Variants class 3 and above were further examined in the published literature and inherited mutation databases including ClinVar (http://www.ncbi.nlm.nih.gov/clinvar), HGMD professional [30], OMIM and locus-specific databases (InSiGHT [28] and Leiden open variation databases [31]) before being assigned a final consensus class. Variants characterised as Class 4 or 5 (pathogenic or likely pathogenic) in genes with established clinical utility were triaged to a Molecular Multidisciplinary Team Meeting (MolMDT). The full body of variant evidence was presented to the MolMDT for evaluation and considered established clinical guidelines. These were then reviewed in the context of the individual patient and a formal report issued documenting evidence-based decision-making (Additional file 4). All cases involved consultation with clinical teams or collaborators prior to dissemination, for guidance where needed and to offer the opportunity to engage with the MolMDT discussion. Participants and clinical teams were followed up in accordance with standard APGI clinical protocols and at intervals post result communication (Fig. 1).

## Results

A total of 463 patients with clinically sporadic PC were recruited to the study between 2009 and 2013. Of the participants, 97.8% (n = 453) indicated via the consent process that they wished to have results returned to them or to their designee. Of the cases, 392 (84%) satisfied project-specific technical and quality essentials, such as minimum sample weight and tumour cellularity, permitting entry into the sequencing pipeline. We performed whole-genome (n = 184) and exome (n = 208) sequencing of matched tumour-normal DNA pairs from eligible patients to an average depth of 65× (tumour) and 35× (germline) under conditions previously described by this group [1, 32, 33]. No patient had an established hereditary cancer syndrome at enrolment. The median age at diagnosis was 67 years and the age range was 33–90 years. A total of 48,904 germline variants were identified, of which 2356 were unique in 130 candidate cancer predisposition genes. Participants had a median of 205 variants each, in the range of 2–633 variants each across the cohort. A total of 568 (24%) variants were ranked as class 3 or above and underwent additional review to assess presence in published literature and inherited mutation databases [30, 34]. Fifty-eight variants (2.5%) in 32 genes remained classified as pathogenic (Class 4 or 5) and were reported in 65 different patients. Upon final classification, 24 variants in genes with established clinical utility were reviewed at the MolMDT. Review, work up and discussion of each variant took an estimated average of 5 working hours of personnel time. Following MolMDT review 19 pathogenic variants reported in 20 cases were recommended for return. The characteristics of cases returned are described in Table 1. Cases with available sample (n = 14) proceeded to independent validation through an accredited laboratory issuing a clinical report and all mutations were successfully validated. In cases where findings could not be validated as no residual germline tissue was available, preliminary results and recommendations from the MolMDT were returned to collaborators to pursue. In keeping with the low survival rate of PC, ten patients (50%) were deceased at the time results were available, four patients (20%) were alive with disease and/or undergoing active treatment or monitoring for their disease, five (25%) patients were discharged from primary specialist care and one (5%) was lost to follow-up.

**Table 1:** Characteristics and outcomes of cases returned

Gene	Variant	Status at time of finding	Dissemination of results	Outcome from dissemination	Clinical utilisation
*ATM*	c.7271 T > G p.Val2424Gly	Alive - with disease. Not undergoing active treatment	Clinician notified	Referral to local cancer genetics service	Family received genetic counselling.
*BRCA2*	c.5410_5411deGT p.Val1804Lysfs*2	Alive - with disease. Undergoing active treatment	Clinician notified	Referral to local cancer genetics service. Clinical utilisation	Family received genetic counselling and offered predictive testing. Switch in treatment to personalised therapy
*BRCA2*	c.4631delA p.Asn1544Thrfs*2	Alive - with disease. Undergoing active treatment	Clinician notified	Referral to local cancer genetics service. Clinical utilisation	Family declined genetic counselling. Switch in treatment to personalised therapy
*CHEK2*	c.1100del p.Thr367Metfs*15	Alive - with disease. Undergoing active treatment	Clinician notified	Clinical decision not to pursue	Nil to date
*PALB2*	c.172_175delTTGT p.Ser59Cysfs*10	Alive - with disease. Undergoing active monitoring	Clinician notified	Referral to local cancer genetics service	Family received genetic counselling and offered predictive testing
*PMS2*	c.1738A > T p.Lys580*	Alive - with disease. Not undergoing active treatment	Clinician notified	Clinical decision not to pursue	Nil to date
*BRCA1*	c.181 T > G p.Cys61Gly	Deceased - of disease	Collaborator notified	No provision for RoR	NA
*BRCA1*	c.3477_3480delAAAG p.Ile1159Metfs*50	Lost to follow-up	Collaborator notified	No provision for RoR	NA
*BRCA1*	c.181 T > G p.Cys61Gly	Deceased - of disease	Collaborator notified	Information resides with collaborators	Nil to date
*BRCA2*	c.7180A > T p.ARg2394*	Alive - without disease. Not undergoing active monitoring	Collaborator notified	No provision for RoR	NA
*BRCA2*	c.7976G > A p.Arg2659Lys	Deceased - of disease	Collaborator notified	Information resides with collaborators	Nil to date
*BRCA2*	c.6702delT p.Phe2234Leufs*7	Alive - without disease. Not undergoing active monitoring	Collaborator notified	Referral to local cancer genetics service	Family received genetic counselling and offered predictive testing
*CDKN2A*	c.344G > T p.Arg115Leu	Deceased - of disease	Collaborator notified	No provision for RoR	NA
*PALB2*	c.3362delG p.Gly1121Valfs*3	Deceased - of disease	Collaborator notified	No provision for RoR	NA
*VHL*	c.154G > T p.Glu52*	Deceased - of disease	Collaborator notified	No provision for RoR	NA
*BRCA2*	c.5239insT p.Asn1747*	Deceased - of disease	Letter direct to patient or next of kin	Referral to local cancer genetics service	Family received genetic counselling and offered predictive testing
*BRCA2*	c.5722_5723delCT p.Leu1908Argfs*2	Alive - with disease. Not undergoing active treatment	Letter direct to patient or next of kin	No reply	Nil to date
*BRCA2*	c.9294C > G p.Tyr3098*	Deceased - of disease	Letter direct to patient or next of kin	No reply	Nil to date
*CDKN2A*	c.146 T > C p.Ile49Ser	Deceased - of disease	Letter direct to patient or next of kin	Referral to local cancer genetics service	Family received genetic counselling and offered predictive testing
*PALB2*	c.3116del p.Asn1039Ilefs*2	Deceased - of disease	Letter direct to patient or next of kin	No reply	Nil to date

Observing the guidelines in the established RoR pathway results were disseminated considering context as follows: directly to the principal collaborator where samples were contributed by third parties (n = 9); through clinical teams where patients were undergoing active treatment or monitoring (n = 6); direct to patient or family members via letter where patients were deceased or no longer receiving active specialist care (n = 5).

Upon follow-up to date, seven cases (35%) have been clinically actioned, defined as consultation and ongoing management within a clinical genetics service, and/or having an influence on clinical decision-making for active clinical care. Specifically, two cases with *BRCA2* mutations were switched to third-line therapy with mitomycin C. The time lapse from result dissemination to action was in the range of 59–626 days.

The residual cases not defined as actioned were categorised as follows: no allowance for RoR in collaborator policy (n = 8); clinical decision not to pursue (n = 2); and non-response to communication (n = 3).

## Discussion

In this report, we describe the longitudinal process of returning germline research results in a cancer context via established mechanisms. These data allow us to illustrate a range of clinical and infrastructural challenges, and practicability, best explored along the complex continuum of RoR. It is to the best of our knowledge the first to report this within a contemporary cancer genomics setting over a protracted period of time.

Overall, 19 pathogenic variants in eight candidate cancer genes in 20 patients were deemed actionable. This represents an actionable finding rate of 5.1% (n = 392) in our cohort. Seven cases were actioned, resulting in 1.78% (n = 392) of the cohort experiencing a clinical benefit from returning research results. Although this number appears low, it is important to note that 63% (n = 7/11), of participants or family members who actually received information, actioned the finding. This is significant given that PC has a high rate of mortality and that 50% of participants were deceased at the time of return. This high rate of action establishes the importance of RoR to participants and family members and the potential benefit it may present.

Reports of actionable finding rates have been shown to be diverse and dependant on the disease setting and specific genes assessed [35, 36]. Moreover, these actionability rates are often theoretically based and defined using variant classification in absence of detail surrounding clinical utility and communicability in the context of the individual patient.

In our experience, the overall process of managing RoR was a resource-demanding activity. The requirements for developing extensive infrastructure required for evaluating genetic findings, disseminating results and performing detailed follow-up was labour-intensive, costly and time-consuming. It also required the input and coordination of a MDT consisting of both research and clinical personnel, including but not limited to: genetic pathologists; clinical geneticists; oncologists; surgeons; genetic counsellors; and bioinformaticians. Significant time and cost considerations were experienced with approximately 5 person-hours on average to review each variant prior to MolMDT, discussion within the MolMDT, independent confirmatory testing, re-contact of next of kin, and recording and disseminating paperwork. Moreover, many of these costs could not be accurately considered from the outset due to lack of guidelines on how to formally manage such a process at the time and the intrinsic unpredictability of RoR. Budgetary and personnel requirements for RoR need to be considered within the context of the individual project at the design stage alongside those for sequencing and analysis.

Due to the diversity of results and of individual patient situations, it was imperative to allow flexibility in the method of communication. Forty percent of cases where notification was by written letter were actioned and lower rates of response to letters is reported [37, 38]. Letters to next of kin were employed as the mode of communication where participants were deceased (55% of the cohort, Table 1), as this represented the only remaining and appropriate outlet to communicate with them. Letters may have been emotionally confronting when received by the next of kin in these situations, especially if they were unaware of the research. Returning results to relatives of deceased participants is not currently widespread practice [39], and while procedures were in place for deceased participant cases, it is recommended that participants are made aware of this possibility and asked to discuss it with their relatives if possible while alive. Australian Privacy law [40] does not address the use of information from deceased persons. However, an important amendment to the privacy act [41] allows for communication with family members without consent of a person under their care if a medical practitioner feels there is a risk of harm to them arising from knowledge about that person. Greater action rates (66%, Table 1) were experienced where treating clinicians were notified and the participants and or family were informed face-to-face by the clinical team. This existing relationship allows for these often-complex discussions to take place in an environment where information can be reinforced and relevance put into perspective for clinical decision-making. Engaging treating clinicians in the MolMDT process prior to dissemination can facilitate a smooth transition into necessary clinical pathways or therapeutic actions, as they are armed with paperwork necessary for referrals of further genetic testing or other relevant supporting information. In summary, our experiences demonstrate that result dissemination needs to be considered in the context of the participant and their family at that specific point in time.

Returning results discovered through the work of international scientific research consortia adds a layer of complexity not routinely experienced by research studies in the past. There is currently no guidance for international consortia, and in most instances the responsibility for RoR is delegated to the local level through consortia developed policies. In our study, 45% of actionable cases were from international collaborators (Table 1) and the applicability of our RoR process was restricted due to collaborators local polices, consent parameters and country-specific guidelines. Only one case could be confirmed as actioned. Our experiences highlight the need for a level of harmonisation of RoR approaches globally. Furthermore, the value and suitability of individually developed consortia-level policies as the primary management tool for RoR will inevitably be bought into question as genome sequencing on a large scale continues to advance. Future approaches could include integration of consortium level oversight while allowing for necessary local autonomy.

Despite the vast amount of effort on review and dissemination of results, the overall number of patients who experienced a clinical benefit from the return of research results in our cohort was low. Furthermore, this low action rate was experienced despite the research being associated with a diverse team of scientific and clinical specialties, regular participant follow-up, high participant and community engagement, a considered budget and experience with producing high quality datasets. Moreover, these findings were mostly related to well-established cancer genes routinely managed in clinical care. Our study highlights how clinicians may be expected to respond to genomic information they had not initiated, but are receiving directly or indirectly through research targeting the tumour genome. Clinicians need to be equipped and empowered to use genomic information in routine practice and concerns about genetic literacy and preparedness for genome sequencing has been voiced by clinicians themselves [42, 43]. Further complexities arise when pathogenic mutations are detected outside of a typical disease phenotype where clear evidence based guidelines for management are less established, as illustrated by the clinical decision not to act in two cases (Table 1). Re-designing a pathway that enables RoR as part of the health system will enhance the interaction across the continuum of research and clinical care and help address the feasibility and economic issues and in turn support sustainability.

While case studies, commentaries and opinion pieces have been useful to date, the RoR process is not static. Application of RoR requires a dynamic longitudinal view of the process incorporated with outcomes. Effectively navigating the RoR process is a current global challenge and significant investment has been recently injected to analyse the ethical, social and legal effects of genomic information [44]. Reports of empirical data are crucial to ascertain the range of benefits and harms of RoR. Plenty of obstacles and challenges no doubt remain, as returning important research information to participants is a young pursuit. The significant investment in overcoming the challenges of sequencing and analysis on a large scale needs to be mirrored in RoR, as the technology of genomic sequencing itself moves into clinical care, application of important research results is fundamental to translational medicine.

## Conclusion

Of our cohort, 5.1% had results that were theoretically actionable. However, only seven cases were actioned, resulting in 1.78% of the cohort of 392 participants deriving clinical benefit from RoR. Despite this low number, 63% of those who received information acted upon this information, demonstrating that RoR stands to be of significant value to participants and family members if we can work to steadily remove the clinical and procedural barriers to implementation.

## Additional files

Additional file 1: APGI Return of Results Guidelines, which outline standard procedures instituted for the return of results process. (PDF 832 kb)

Additional file 2: Gene List, which includes the list of candidate genes selected for the RoR process. (XLSX 60 kb)

Additional file 3: Variant Classification Schema, which outlines the bioinformatic and final classification ranking for assessment of variants. (PDF 271 kb)

Additional file 4: Molecular MDT Protocol, which outlines standard operating procedures for the MolMDT. (PDF 271 kb)

## Abbreviations

MolMDT: Molecular multidisciplinary team meeting
PC: Pancreatic cancer
RoR: Return of results
