# Supplementary material for: Lost in translation: returning germline genetic results in genome-scale cancer research
Source: Genome Med. 2017 Apr 28;9:41. doi: 10.1186/s13073-017-0430-4 (PMC5408494; doi:10.1186/s13073-017-0430-4)
Supplement: Supplementary file 1 — APGI Return of Results Guidelines, which outline standard procedures instituted for the return of results process. (PDF 832 kb) [file 13073_2017_430_MOESM1_ESM.pdf]

Guidelines on Returning Individual Research Results (RoR) to Individuals
Interpretation, Review and Action of Data

1. Background

Over the last decade, next-generation sequencing technology (NGS) has developed to the point that rapid and relatively affordable sequencing of individual genomes is a reality. NGS offers the promise of tremendous public benefit as it underpins both improvements in our understanding of disease as well as substantially changing our practical ability to translate this knowledge through improved diagnostics and therapeutics. The advent of NGS has also seen a parallel increase in the debate around whether and how to disclose individual results of genetic sequencing to research participants. Whether researchers have a moral obligation to provide findings of this nature back to research participants is a vexed issue with no international consensus. The matter is complicated by the fact that unlike clinically validated genetic tests, WGS and other high throughput research techniques are research tools – they are not designed for clinical diagnosis and they may produce results that are of questionable clinical utility. Results from these analyses may identify genetic variants that are related to increases in disease risk, but the increased risk may be exceptionally small. Nevertheless, findings that are currently uncertain may, in future, become clinically relevant, and on this basis that the APGI has considered this issue at length, and concluded that within the context of a robust framework and close consultation with clinical teams, returning results to participants is feasible and should be conducted whenever indicated.

2. Key RoR Principles

- Important research results are often referred to under various designations, such as incidental or secondary findings, off target results etc, and are used interchangebly throughout literature and policy.
- These guidelines and all policies and procedures related to RoR are approved by the APGI leadership team (http://www.pancreaticcancer.net.au/apgi/leadership-teams).
- The point of policy reference for these guidelines is NHMRC National Statement on Ethical Conduct in Human Resaerch 2007.
- It is crucial that each result is assessed on a case by case basis, carefully considering context along with the significance of the result.
- Managing RoR is a shared responsibility between researchers and clinical teams, and at all times where appropriate researchers should work with clincial teams to ensure information is handled appropriately and in-line with the ethically defensible plan described herein.
- RoR is an opt-in situation for the APGI participants, which is addressed through the consent process and recorded through CanSto_Pancreas.
- These guidelines are not all-encompassing, as every situation that arises will be varied. Procedures presented herein are designed to be iterative and updated every 6 months (or at every 2nd ICGC data release).

3. Ethically Defensible Plan

The Australian National Statement on ethical conduct in Human Research (2007) is designed to clarify repsonsibilities of insitutions and researchers for the ethical design, conduct and dissemination of results of human research. Return of results is addressed in section 3.5.1, where it states: "Where research may discover or generate information of potential importance to the future health of participants, or their blood relatives, researchers must prepare and follow an ethically defensible plan to disclose or withhold that information". The APGI has developed an Ethically Defensible Plan (EDP), which has been approved by all HREC's at active APGI sites. The EDP framework set out by the APGI for returning results employs a context-dependent paradigm, and enacts a broad category-based system for the characterisation of research findings. The framework was developed as an iterative, evidence-based and consensus driven process, with engagement of key stakeholders. As the primary research indication is the interrogation of cancer genomes for the discovery and analysis of driver mutations, the return of results is limited to findings related to cancer.

The key components of the plan are:

1. Consent

Through the consent process, participants are provided with information in relation to the fact that relevant findings may be discovered in the course of the study, and that these may not be limited to pancreatic cancer. The consent process allows participants the choice to "opt in" to have individual results communicated, and given the short survival of many individuals with pancreatic cancer, the choice of to whom else they may be communicated to. It is usual practice during the consent process to obtain contact details of a family member or significant other who is identified to receive information. Whilst participation of family members in the consent process is typical and was encouraged for the above reasons, relatives are not considered formal participants of the study and explicit consent was not required under this protocol (upon guidance from our approving HREC's). Participants are informed that any information would initially be discussed with their treating doctor, and their preference for returning results is logged and tracked through a research database.

2. Significance of Results

a. Candidate Genes

Genes were selected that were deemed by the APGI Leadership Team to be important, implicated and or associated with cancer in general. The list includes genes with established risk for PC, genes with a cancer-related syndrome or a well-characterised solid organ or haematological cancer phenotype. Currently only a small number of these would be considered to have clinical utility. Given the research nature of our work, our intended scope was to be inclusive, to maximise the opportunity to further interrogate as many genes as possible to further investigate their involvement in cancer. The full candidate gene list is is outlined in Appendix 1.

b. Classification System and Review

A broad based categorical system is enacted, based on the below:

I. ***Pathogenic*: Class 5**

Variant is previously reported, and pathogenic in terms of found to contribute mechanistically to disease, but is not necessarily fully penetrant (ie may not be sufficient in isolation to cause disease). Examples of consequence/variant types often represented in this category include: transcript ablations, splice variants (donor or acceptor; essential splice site variant), stop gained (premature stop codon) or frameshift variant.

These findings represent direct utility, in terms of disease prevention, or where a medical benefit exists of a prognostic, diagnostic or therpeautic nature. These types of findings often have established treatment guidelines and confer a high risk of a preventable disease. The majortity of results falling into this category are germline suspectibility mutations, but also exemplifies somatic mutation information that may indicate a discordant pathology diagnosis. These findings are always confirmed in an indepedant diagnostic grade assay (see below for detailed information on *Validation of Results*). Variants in this category are always tabled for review at the Molecular MDT Meeting.

II. ***Likely Pathogenic: Class 4***

Variant maybe previously unreported, but is of the type which is expected to cause the disorder or disease. These findings may also be previously reported, but may not have established management guidelines in place to adequately guide surveillence or future management. Examples of variant types often represented in this category include: stop lost variants, non-synonymous coding variants including: initiator codon, inframe insertions or deletions. Current examples within the context of this project are germline *PALB2* and *CHEK2* mutations, which have been shown to be pathogenic but do not have established management procedures in routine clinical practice. These findings are confirmed in an independent diagnostic grade assay (or equivalent) wherever possible, and if availability of confirmatory testing is in question, a clinical geneticts should be consulted for advice on what levels of confirmatory testing are acceptable. Variants in this category are also always tabled for review at the Molecular MDT Meeting.

III. Uncertain significance: Class 3

Class 3 variants all have a minor allele frequency <0.01 and include in-frame indels, splice region variants and missense variants with predicted deleterious consequence according to VEST3 or conflicting reports of pathogenicity.

IV. Probably no pathogenicity: Class, Benign/No Pathogenicoty: Class 1

Class 1 and class 2 variants are comprised of missense variants which either have a MAF < 0.01 but are predicted to be benign or a MAF ≥0.01, synonymous variants and non-coding variants (intronic, UTR, down- and up-stream).

Clinical Utility

Variants were considered actionable where clinical utility was established through the MolMDT, with regard to prevention, diagnosis, prognostication and or therapy.

There is an additional layer to clinical actionability in cancer: where variants are previously not known to be causative, but associated with a clinical presentation. These findings have demonstrated clinical validity, but lack demonstrated clinical utility in a current treatment setting. They also may be members of cancer pathways or functional groups that are targets for approved or investigational therpeautic agents or biomarkers. Clinical validity may refer to: significant evidence in literature, functional evidence, previously published reports. These findings are always reported with a brief explanation of their relevance to pancreatic cancer, inlcuding providing literature or associated data where applicable. Other important factors are also often considered such as clinical presentation, individual risk, family history and clinical relevance.

3. Communicability & delivery of results

Communicability considers the practicality of communicating results, the circumstances of the patient and treating clinician, while delivery of results considers how best the results could be communicated. This is an important point to consider in sensitive situations such as particpants being deceased. Where participants are alive and undergoing active treatment or monitoring of their disease, findings are communicated to their treating clinical team, whom would triage the information to participants or family members. Where participants may be deceased or not undergoing active clincal care, they will be communicated either directly if alive, or to their nomincated NOK or designee if deceased (as outlined in the consent process). As approaches to RoR differs globally, if patients are contributed by international collaborators it is standard process to communciate results directly to the prinicpal investigator for confirmation and consideration as appropriate in their jurisdiction.

This ethically defensible plan is based on beneficence, reciprocity and respect for participants, which are all fundamental ethical principles in research. On contact by the treatment team, participants have the option not to pursue further information, which values the autonomy and voluntary nature of research participation.

4. Review and Interpretation of Variants

Data is received by the APGI review team as High Confidence Calls, which are received in batches directly from the UQ private Cluster. Variants are considred high confidence when they are identified by both of the pipeline's variant callers, qSNP and GATK. The genomic coordinates of germline variants were annotated for gene consequence with ensembl v75. The effect of missense variants were predicted using polyphen2 SIFT, CADD, VEST3, alignGVGD, MutationTaster, phyloP and phastCons. Allele frequency in the general population was obtained from the 1000 Genomes Project (www.1000genomes.org), Exome Aggregation Consortium (http://exac.broadinstitute.org/about) and dbSNPv141 (http://www.ncbi.nlm.nih.gov/SNP).

All mutations falling into category 1 or 2, and all missense SNVs that were deemed deleterious using bioinformatic tools (VAST3) (need ref), or where qualitative scores indicate the finding was deleterious or damaging, are further investigated using inherited disease mutation databases (ref Stenson- HGMD) and literature review. This data is collated and presented to the monthly MDT meeting (further details outlined below). After MDT review and discussion, those findings being considered for return are then sent for confirmatory testing using a diagnostic grade assay.

Other Considerations

1. **Validation of Results and Confirmatory Testing**
   Although results may proceed to MDT review, prior to any results being officially returned, they must be confirmed or validated in an independent, preferrably diagnostic grade test. Most highly penetrant germline mutations have NATA diagnostic grade tests that are routine and easily accessible. Currently availalble genetic tests throughout Australia can be found at the RCPA's Catalogue of genetic tests and laboratories (http://genetictesting.rcpa.edu.au/).
   Other variants being considered for return may not have an approved diagnostic grade test available, and in these situations a suitable confirmatory test must be persued prior to return. For example, a clincial grade, accredited department or institution may offer targeted sanger seqeuncing for a particular area, but the report may not be considered diagnostic grade. This will at times be considered suitable and reduces the chances of error in the research sample. Validating with a non-NATA assay but in a NATA lab is still sufficient in many cases where this is standard clinical practice. Practical information on how to submit a sample for confirmatory testing can be found in the APGI's *Confirmatory Testing Guidelines* Manual.

2. **Databases**:
   Review and interpretation must take place using multiple lines of evidence, and the extent to which a variant may be considered important may be influenced by a number of various other parameters, particularly those which may fall into the categories of potential clinical utility or findings with personal meaning. Variants often undergo further review using multiple sources. The main databases consulted are locus specific databases such as HGMD, Alamut and LOVD. HGMD is accessed via the APGI group subscription for all MDT members. For further advice on using HGMD or Alamut please refer to the *APGI's Database Applications Manual*. Disease specific databases or data sets such as KConfab and Insight may also be consulted.

5. Multi-disciplinary Review (Molecular MDT)

As the volume of somatic and germline genomic data produced by our sequencing efforts was rapidly increasing, it was recognised that a multidisciplinary team was required to discuss significant molecular findings and make evidence- and consensus-based recommendations on how to manage the information. The aims are:

1. To review and discuss somatic and germline genomic research data that is presented as reportable, actionable, novel and/or interesting
2. To build evidence via literature reviews, current clinical guidelines and genome databases and determine appropriate course of validation

3. To formally document recommendations for each case discussed, and when appropriate, report these back to the treating clinician
4. To review pancreas patients screened and deemed eligible for the IMPACT clinical trial, due to the presence of clinically actionable variants.

Terms of reference and formal process is outlined below:

- The MDT/discussion panel consists of a unique combination of clinical and research expertise to determine the best possible course of action for significant research findings who meet on a monthly basis, or more frequent during busy periods of data relsase.
- It consists of a core group of attendees including:medical oncologists physcician/scientist, Gastroenterologist physician/scientist, genetic pathologist, project manager and a genetic counscellor. A second teir of members contribute on a consultative basis when needed, including a genomicist, informatician, clinical geneticist and anatomical pathologist.
- An agenda is circulated so that members can assess their degree of input for the upcoming discussions. Proposed cases will be submitted via email to the meeting coordinator (s.simpson@garvan.org.au) outlining the genomic finding, verification and validation processes and preliminary evidence. Patient history is then extracted from the clincial database and added.
- At the start of each meeting, one volunteer presents a piece of applicable and current literature, including best practice guidelines and case studies (approximate time of 10 mins).
- The criteria for submitting a variant for discussion is that (as outlined above) the data be verified and has undergone IGV review. The loci and nature of the variant is assessed as outlined above, and all variants falling into category I,II or III are welcomed to the MDT. Higher priority is given to suspected category I varaints.
- After presentation of each case and its applicable evidence, a decision is made upon reaching 80% consensus of the group. If 80% concensus is not reached, further review may be undertaken by other specialist teams or consultative networks. Given all of our candiate genes are cancer related, this is an infrequent occurance.
- Report templates will be prepared so that the recommendations/opinions of the panel, including the category of the molecular variant, can be officially documented. Consideration will also be given to how communicable specific results are, and possible triage with other clinical services. These reports are also often appended to the official mutation notification letter, to advise clinical teams of the chain of custody undertaken prior to the reporting of the finding.
- Reports and any other relevant documentation are uploaded to the pateints case file in CanSto_Pancreas.
