# Supplementary material for: Lost in translation: returning germline genetic results in genome-scale cancer research
Source: Genome Med. 2017 Apr 28;9:41. doi: 10.1186/s13073-017-0430-4 (PMC5408494; doi:10.1186/s13073-017-0430-4)
Supplement: Supplementary file 3 — Variant Classification Schema, which outlines the bioinformatic and final classification ranking for assessment of variants. (PDF 271 kb) [file 13073_2017_430_MOESM3_ESM.pdf]

Variant Classification Schema

Variant ranking	Initial bioinformatic classification	Final classification	
**Class 5 = pathogenic**	Genomic coordinates fed to VEP on ENSEMBL 75:<br>"splice_donor_variant"<br>"splice_acceptor_variant"<br>"stop_gained"<br>"frameshift_variant"	Transcript abrogating variants (nonsense, frameshift, consensus splice site) with previous reports of pathogenicity	Missense variants with functional characterisation demonstrating a functional effect relevant to the disease phenotype and multiple independent reports of pathogenicity.
**Class 4 = likely pathogenic**	"stop_lost"<br>"initiator_codon_variant"<br>"inframe_insertion"<br>"inframe_deletion"<br>"missense variant" and 1000 genomes project combined MAF < 0.01 and VEST3 call deleterious	Previously unreported variants predicted to lead to protein truncation (nonsense, frameshift, consensus splice site, initiator codon, non-stop). Variants predicted to abrogate the transcript but occurring in the last exon were called class 3 unless a functional effect or pathogenicity had been previously demonstrated.	Missense variants with supporting functional evidence, but lacking multiple independent reports of pathogenicity.
**Class 3 = uncertain significance**	MAF < 0.01 and<br>"splice_region_variant"<br>"incomplete_terminal_codon_variant"<br>"missense variant" and 1000 genomes project combined MAF < 0.01 and VEST3 call not available	Class 3 variants all have a minor allele frequency <0.01 and include in-frame indels, splice region variants and missense variants with predicted deleterious consequence according to VEST3 or conflicting reports of pathogenicity.	
**Class 2 = probable non-pathogenic**	1000 genomes combined MAF > 0.01 and/or "missense variant" and VEST3 call tolerated	Class 1 and class 2 variants are comprised of missense variants which either have a MAF < 0.01 but are predicted to be benign or a MAF ≥0.01, synonymous variants and non-coding variants (intronic, UTR, down- and up-stream).	
**Class 1 = non-pathogenic**	"synonymous_variant"<br>"stop_retained_variant"<br>"coding_sequence_variant"<br>"5_prime_UTR_variant"<br>"3_prime_UTR_variant"<br>"non_coding_exon_variant"<br>"nc_transcript_variant"<br>"intron_variant"<br>"NMD_transcript_variant"<br>"upstream_gene_variant"<br>"downstream_gene_variant"<br>"TFBS_ablation"<br>"TFBS_amplification"<br>"TF_binding_site_variant"<br>"regulatory_region_variant"<br>"regulatory_region_ablation"<br>"intergenic_variant"		
