# Supplementary material for: Lost in translation: returning germline genetic results in genome-scale cancer research
Source: Genome Med. 2017 Apr 28;9:41. doi: 10.1186/s13073-017-0430-4 (PMC5408494; doi:10.1186/s13073-017-0430-4)
Supplement: Supplementary file 4 — Molecular MDT Protocol, which outlines standard operating procedures for the MolMDT. (PDF 271 kb) [file 13073_2017_430_MOESM4_ESM.pdf]

APGI Molecular MDT Protocol

1. BACKGROUND

The Australian Pancreatic Cancer Genome Initiative (APGI) generates diverse germline and somatic genomic data on hundreds of pancreatic cancer patients participating in research. The APGI is committed to returning individual clinically significant research results to participants and has implemented guidelines to ensure key components of the return of results (RoR) process are approached consistently and in line with current recommendations (see APGI Guidelines on Returning Individual Research Results to Individuals).

The Molecular MDT is an integral part as it regularly convenes APGI researchers and clinicians with particular expertise to discuss and interpret a subset of genomic variants and consider RoR in the context of the individual participant. Decisions on pathogenicity, clinical significance and communicability are made on an evidence-based and case-by-case approach and formally documented to ensure a transparent decision-making process.

2. METHODS

2.1 Variant Classification and Prioritisation

High-confidence single nucleotide variants (SNVs) or indels are annotated using genomic coordinates and classified according to *in silico* predicted effect of nucleotide substitutions and population allele frequency. Classification is based on an adapted 5-tiered schema [1,2] where Class 5 = pathogenic, class 4 = likely pathogenic, class 3 = uncertain significance, class 2 = likely probable non-pathogenic, class 1 = non-pathogenic (Appendix 1). Variants classified ≥3 undergo manual curation of databases and literature to ensure criteria are met.

Variants classified as pathogenic in well-established and actionable cancer associated genes are prioritised for Molecular MDT discussion. Novel abrogating variants, those with conflicting evidence and Class 4 and 5 variants detected in less well established cancer associated genes are on the next tier for discussion.

2.2 Molecular MDT Case Report

A formal report is prepared for each participant where a suspected clinically significant variant is identified (Appendix 2). There are 5 mains sections to be completed with the following information, where available:

1. <u>Participant Clinical History</u>
   Gender; Status (Alive with/without disease, Deceased of disease); Age at diagnosis; Date and hospital location of resection; Pathology; Treatment history; Primary treating clinicians; Personal and family history of malignancy; Level of consent provided, including next of kin nomination; Availability of DNA stocks

2. Variant Details
   Various nomenclature (genomic coordinates, nucleotide and amino acid level); Consequence (e.g. frameshift, missense, splice site etc.); Current classification (Class 5-1); Sequencing depth (whole genome, exome, panel, targeted); Previous reports and classification in mutation databases (e.g. ClinVar, HGMD Professional, InSiGHT, LOVD, BIC); Links to primary and supporting literature

3. Proposed Actions
   Additional genomic data that may be useful for further decision-making (e.g. somatic data or functional evidence); Accredited laboratory details and sample specifications for external validation; Scientific or clinical contacts for additional expert advice

4. Summary
   Date of meeting; Final consensus variant classification; Justification of no RoR; Action plan if RoR.

5. Outcome
   Progress and outcomes of each case are chronologically documented here.

2.3 Molecular MDT Meeting

The Molecular MDT working group includes research and clinical personnel with particular expertise, such as genetic pathologists, medical oncologists, pathologists, bioinformaticians and genetic counsellors. The group meets in person on a monthly basis for 1-2 hours, with a maximum of 3 cases discussed each meeting. The Case Reports (Sections 1-3) are circulated one week prior, to provide the opportunity for familiarization and independent literature review as allocated.

The minutes for each case are documented in Section 4 of the Case Report. The final variant classification is decided upon once all evidence is presented. The clinical significance and personal utility of the variant is assessed in the unique context of the participant. If the consensus recommends returning the result, an action plan is outlined including independent validation and recommended strategy for result communication (i.e. directly to clinician, participant or next of kin). If no further action is deemed, this rationale is clearly stated in Section 4 of the Case Report.

Updates of cases with results to return are provided in subsequent Molecular MDT meetings and recorded in Section 5. Case Reports are attached to the participant's research file.

3. REFERENCES

1. Richards S, Aziz N, Bale S, et al. Standards and guidelines for the interpretation of sequence variants: a joint consensus recommendation of the American College of Medical Genetics and Genomics and the Association for Molecular Pathology. Genetics in Medicine 2015;17:405-23.
2. Thompson BA, Spurdle AB, Plazzer J-P, et al. Application of a 5-tiered scheme for standardized classification of 2,360 unique mismatch repair gene variants in the InSiGHT locus-specific database. Nat Genet 2014;46:107-15.

4. APPENDICES

Appendix 1 – Variant Classification Schema

Variant ranking	Initial bioinformatic classification	Final classification	
**Class 5 = pathogenic**	Genomic coordinates fed to VEP on ENSEMBL 75:<br>"splice_donor_variant"<br>"splice_acceptor_variant"<br>"stop_gained"<br>"frameshift_variant"	Transcript abrogating variants (nonsense, frameshift, consensus splice site) with previous reports of pathogenicity	Missense variants with functional characterisation demonstrating a functional effect relevant to the disease phenotype and multiple independent reports of pathogenicity.
**Class 4 = likely pathogenic**	"stop_lost"<br>"initiator_codon_variant"<br>"inframe_insertion"<br>"inframe_deletion"<br>"missense variant" and 1000 genomes project combined MAF < 0.01 and VEST3 call deleterious	Previously unreported variants predicted to lead to protein truncation (nonsense, frameshift, consensus splice site, initiator codon, non-stop). Variants predicted to abrogate the transcript but occurring in the last exon were called class 3 unless a functional effect or pathogenicity had been previously demonstrated.	Missense variants with supporting functional evidence, but lacking multiple independent reports of pathogenicity.
**Class 3 = uncertain significance**	MAF < 0.01 and<br>"splice_region_variant"<br>"incomplete_terminal_codon_variant"<br>"missense variant" and 1000 genomes project combined MAF < 0.01 and VEST3 call not available	Class 3 variants all have a minor allele frequency <0.01 and include in-frame indels, splice region variants and missense variants with predicted deleterious consequence according to VEST3 or conflicting reports of pathogenicity.	
**Class 2 = probable non-pathogenic**	1000 genomes combined MAF > 0.01 and/or "missense variant" and VEST3 call tolerated	Class 1 and class 2 variants are comprised of mmissense variants which either have a MAF < 0.01 but are predicted to be benign or a MAF ≥0.01, synonymous variants and non-coding variants (intronic, UTR, down- and up-stream).	
**Class 1 = non-pathogenic**	"synonymous_variant"<br>"stop_retained_variant"<br>"coding_sequence_variant"<br>"5_prime_UTR_variant"<br>"3_prime_UTR_variant"<br>"non_coding_exon_variant"<br>"nc_transcript_variant"<br>"intron_variant"<br>"NMD_transcript_variant"<br>"upstream_gene_variant"<br>"downstream_gene_variant"<br>"TFBS_ablation"<br>"TFBS_amplification"<br>"TF_binding_site_variant"<br>"regulatory_region_variant"<br>"regulatory_region_ablation"<br>"intergenic_variant"		

Appendix 2 – Molecular MDT Case Report

Patient ID: **APGI_**

PATIENT HISTORY

Include gender, status, residing state/country, age at diagnosis, surgery details, pathology, chemotherapy, personal or family history of cancer, level of consent given, any NOK and available biospecimens

-
-
-

MOLECULAR FINDINGS

Outline various nomenclature, predicted effect (e.g. missense, frameshift), database findings, supporting literature and proposed variant category (e.g. clinically significant, unknown clinical significance)

WGS WES Panel *(circle one)*

**Gene (Germline or Somatic)**	**Variant**	**HGMD**	**dbSNP/ClinVar**	**LOVD**
	g.<br>c.<br>p.			

Literature and Evidence:

PROPOSED ACTIONS

List action items and key issues if results were to be returned/not returned. Include details on laboratories for molecular validation and any specialist research/clinical staff recommended or approached for input

-
-

MDT SUMMARY

Outline the plan forward, those responsible and any key discussion points. Please note the date of the meeting

Date:

-

OUTCOME

If the result was returned, summarise who returned the result, when and how the result was returned and what the outcome was

Date:
Date:
Date:
